# Selection and validation of reliable reference genes for gene expression studies from *Monilinia vaccinii-corymbosi* infected wild blueberry phenotypes

**DOI:** 10.1038/s41598-020-68597-9

**Published:** 2020-07-16

**Authors:** Sherin Jose, Joel Abbey, Laura Jaakola, David Percival

**Affiliations:** 1 Wild Blueberry Research Program, Faculty of Agriculture, Dalhousie University, Truro, NS B2N 5E3 Canada; 2 Climate Laboratory Holt, Department of Arctic and Marine Biology, The Arctic University of Norway, 9037 Tromsø, Norway; 3 NIBIO, Norwegian Institute of Bioeconomy Research, P.O. Box 115, 1431 Ås, Norway

**Keywords:** Plant molecular biology, Plant stress responses, Plant sciences

## Abstract

Monilinia blight disease caused by *Monilinia vaccinii-corymbosi* (Reade) Honey (*M.vc*) causes severe damage and economic losses in wild blueberry growing regions. Molecular mechanisms regulating defence responses of wild blueberry phenotypes towards this causal fungus are not yet fully known. A reliable quantification of gene expression using quantitative real time PCR (qPCR) is fundamental for measuring changes in target gene expression. A crucial aspect of accurate normalisation is the choice of appropriate reference genes. This study evaluated the expression stability of seven candidate reference genes (*GAPDH*, *UBC9*, *UBC28*, *TIP41*, *CaCSa*, *PPR* and *RH8*) in floral tissues of diploid and tetraploid wild blueberry phenotypes challenged with *M.vc.* The expression stability was calculated using five algorithms: geNorm, NormFinder, BestKeeper, deltaCt and RefFinder. The results indicated that *UBC9* and *GAPDH* were the most stable reference genes, while *RH8* and *PPR* were the least stable ones. To further validate the suitability of the analyzed reference genes, the expression level of a pathogenesis related protein gene (i.e., *PR3*) was analysed for both phenotypes at four time points of infection. Our results may be beneficial for future studies involving the quantification of relative gene expression levels in wild blueberry species.

## Introduction

Monilinia blight caused by *Monilinia vaccinii-corymbosi* (Reade) Honey, is a destructive disease in commercial wild blueberry fields in the Atlantic Provinces of Canada and Maine, US. Under favourable weather conditions, infections may cause considerable losses in yield and post-harvest quality of berries^1,2^. The pathogen infects almost all blueberry species, such *as V. angustifolium* (sweet lowbush blueberry), *V. myrtilloides* (sour top lowbush blueberry), and *V. corymbosum* L. (northern highbush blueberry). The wild blueberry fields comprise tetraploid, *V. angustifolium*, *V. a*. f. *nigrum*, and diploid, *V. myrtilloides* phenotypes. Commercial wild blueberry fields mostly consist of *Vaccinium angustifolium* (tetraploid) (~ 70–80% on a surface area basis), *V. myrtilloides* (diploid) (~ 10–20%) and a few hybrids^3^.

Monilinia damage is often variable in commercial fields due to varying levels of genotypic resistance, differences in morphological features, and bud growth and development among phenotypes. Over the years, minimal damage from monilinia blight has been reported in *V. myrtilloides* and has been identified as the potential source of Monilinia blight resistance^4,5^. In addition to genetic and morphological variability, sufficient variations in yield parameters (berry size, number of berries per cluster, stem density) have been reported between the two phenotypes^6^. Although the *Monilinia–Vaccinium* pathosystem is an important phenomenon, a detailed molecular understanding of gene expression profile during the infection process is lacking within and between the phenotypes in wild blueberries.

Analyzing stress responsive genes from diverse biological samples under experimental conditions provides insights into the underlying signalling and regulatory mechanisms^7^. Quantitative real time PCR (qRT-PCR) has become an effective approach to examine and validate the changes in gene expression owing to its accuracy, specificity and sensitivity^8–11^. However, the reliability of the technique is largely influenced on the normalization strategy such as the usage of one or more stable reference genes. Ideally, the reference genes used for normalisation should have a uniform expression regardless of the experimental conditions^11,12^. Hence, the selection and validation of stable reference genes for each experimental condition is a prerequisite for performing qRT-PCR studies^13–15^.

In the present study, seven reference genes (*GAPDH, CaCSa, TIP41, UBC28, PPR, UBC9* and *RH8*) were selected as candidate reference genes based on previous reports in *Vaccinium* spp^16,17^. Their expression stabilities in floral tissues of diploid and tetraploid wild blueberry phenotypes challenged with *Monilinia vaccinii- corymbosi* was evaluated at four time-points after infection. Five different statistical software programs (geNorm^8^, NormFinder^18^, BestKeeper^19^, delta Ct^20^ and RefFinder^21^) were used to analyse the stability of the candidate reference genes and to select the most appropriate ones. This study will lay a foundation for future gene expression research in wild blueberry.

## Results

### Selection of reference genes, amplification efficiency and specificity

A total of seven candidate reference genes were selected to identify suitable RGs for gene expression studies using qPCR in wild blueberry. Additionally, *PR3* gene was used to validate the accuracy of identified RGs in wild blueberry phenotypes infected with *M. vaccinii-corymbosi*. The specificity of the analysed primer pairs was confirmed via detecting single fragment of the expected size on 2% agarose gel electrophoresis and a single peak with no signals on the negative controls in the melt curve analysis (Table 1, Supplementary Fig S1 & S2). All the tested RGs had efficiency (E %) values ranged from 95 to 105%, with regression coefficient (R^2^) varying from 0.994 to 0.999 (Table 1, Supplementary Fig S3). The results showed that all the primer pairs were suitable for RT-qPCR analysis.

**Table 1:** Candidate reference genes analysed in the study and parameters derived from RT-qPCR analysis.

Sl no.	Gene name	Gene description	Gene ID	Primer sequence (5′–3′)	Amplicon size (bp)	Annealing Tm (°C)	Primer efficiency (%)	Regression coefficient (R^2^)	References
1	GAPDH	Glyceraldehyde 3-phosphate dehydrogenase (GAPDH)	AY123769	CAAACTGTCTTGCCCCACTT	207	55	98	0.998	Koskimäki et al., 2009
				CAGGCAACACCTTACCAACA					
2	CaCSa	Clathrin adapter complexes medium subunit (cacsa)	DR067098	CTGTTGGATGGCGAAGAGAG	98	55	99	0.996	This study
				TTTCCCAGTCACATCACAGC					
3	UBC28	Ubiquitin-conjugating enzyme (UBC28)	CF811189	CCATCCACTTCCCTCCAGATTATCCAT	164	62	97	0.999	Vashisth et al., 2011
				ACAGATTGAGAGCAGCACCTTGGA					
4	RH8	RNA helicase-like (RH8)	DR067965	GGGATAGACATTCAAGCAGTCA	81	55	95	0.996	This study
				ACCAACCCTGTGCAGATAAG					
5	UBC9	SUMO-conjugating enzyme (UBC9)	^a^AT4G27960	CACCCGAATATAAACAGCAATGG	91	55	99	0.997	This study
				ACAGCAACACCTTGGAGATAG					
6	PPR	Pentatricopeptide repeat-containing protein (PPR)	^a^AT1G62930	GGCTTAGTAGAGAAGGGAAGATTG	95	56	105	0.994	This study
				GATATTATACGAGACGGCGTTAGG					
7	TIP41	TIP41-like protein (TIP41)	^a^AT4G34270	TGCCAA GTT CAT GGT TTG TTC T		56	101.4	0.996	This study
				CATACGCGTGTCTCTCAATCTCA	80				
**Target gene**									
1	PR3	Pathogenesis related protein	MK292725	TGTGCTCCTGGGAAGAAGTA	112	55	100	0.998	This study
				AGTCTGGGTTGGCTAGTAGAT					

^a^Arabidopsis homolog locus.

### Expression profiling of candidate reference genes

**Figure 1:**
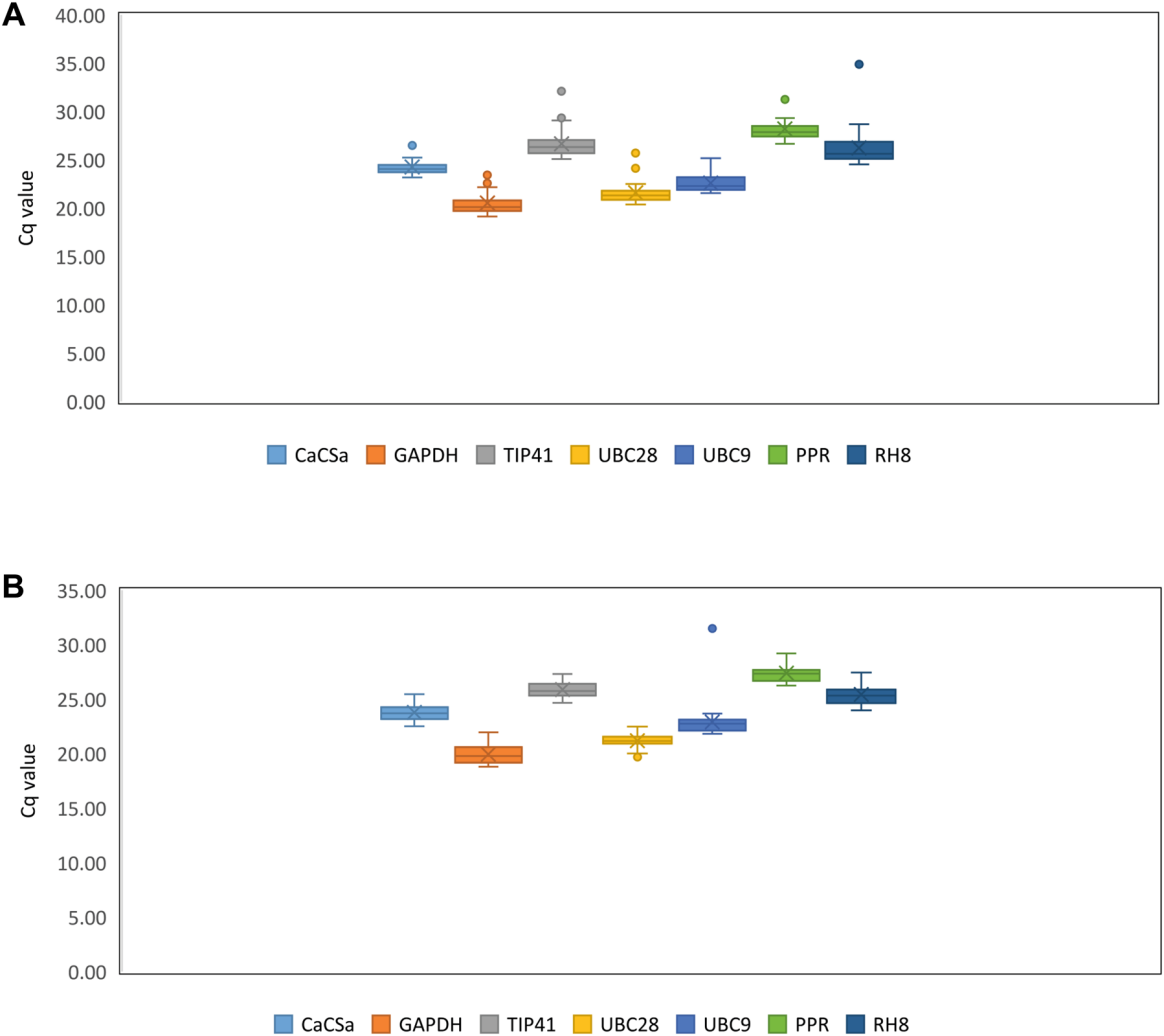
Cq values distribution of seven candidate reference genes in **(A)** *V. a*. f. *nigrum* and **(B)** *V. myrtilloides*. Whiskers represent the maximum and minimum value while the box indicates the 25 and 75th percentiles and line across the box indicates the median.

The raw quantification cycle (C_q_) values were used to quantify the expression levels of candidate reference genes where lower Cq values mean higher expression levels. Cq values for each of the seven candidate reference genes in *V. myrtilloides* and *V. a*. f*. nigrum* are listed in Supplementary Table S1 (there were no Cq values in the negative controls), and a box and whiskers plot were used to describe the raw Cq value distribution (Fig. 1). In *V. myrtilloides*, the C_q_ values varied from 19.03 to 26.65, while it was 18.81 to 29.18 in *V. a.* f*. nigrum*. *GAPDH* was the most expressed gene in both phenotypes with a mean of 19.94 and 20.32 respectively. The stability was analysed in comparison with the time-course of disease infection.

### Stability ranking of the candidate reference genes

Expression stabilities of the seven candidate reference genes were determined using geNorm, NormFinder, ΔCq, and BestKeeper and their overall stabilities were ranked by RefFinder across all the timepoints and phenotypes.

#### geNorm analysis

**Figure 2:**
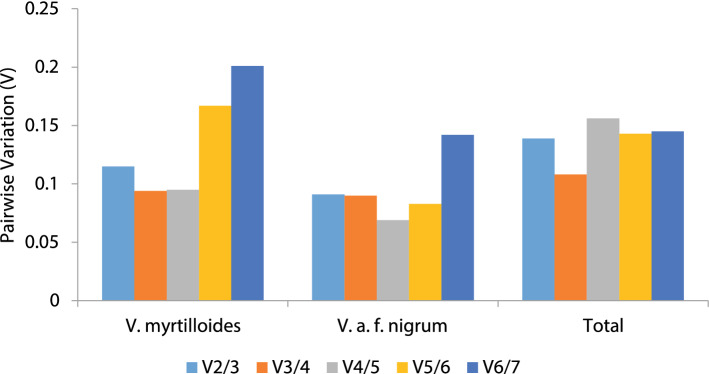
Pairwise variation (V) of candidate reference genes, as calculated by geNorm software for *V. myrtilloides*, *V. a*. f. *nigrum* and total samples. Vn/Vn + 1 values were used to determine the optimal number of reference genes (with threshold value = 0.5).

The expression stability rankings based on the M-values for the seven candidate reference genes on wild blueberry phenotypes is displayed (Table 2). The gene with the lowest M-value (cut-off 1.5) was the most stable reference gene in terms of gene expression and vice versa. The M-values for the tested genes in all samples and groups were lower than the default limit of 1.5. Genorm analysis for *V. myrtilloides* illustrated that *CaCSa* (0.385) and *GAPDH* (0.386) were the more stable genes and *PPR* (0.888) exhibited the least stability. On contrary, *UBC9* (0.239) and *GAPDH* (0.239) exhibited high expression stability in *V. a*. f. *nigrum* and *RH8* (0.432) were the least stable one (Table 2). Among the total samples, *GAPDH* and *UBC9* were the most stable genes with M values of 0.487 and 0.512, whereas RH8 exhibited least stability (0.701). Finally, the pairwise variation (V_n_/V_n+1_) for both the phenotypes and entire group resulted in V_2/3_ < 0.15 (Fig. 2) which indicated that two reference genes were sufficient for accurate normalisation of RT-qPCR data.

**Table 2:** The stability ranking of candidate reference genes of analysed samples from *V. myrtilloides* and *V. a.* f. *nigrum* based on geNorm, NormFinder, BestKeeper, Delta Cq and RefFinder.

Phenotype	Rank	GeNorm		ΔCq		NormFinder		BestKeeper		RefFinder	
		Gene	M	Gene	SD	Gene	SV	Gene	r	Gene	GM
*V. myrtilloides*	1	CaCSa	0.385	UBC28	0.16	CaCSa	0.043	CaCSa	0.62	CaCSa	1.41
	2	GAPDH	0.386	CaCSa	0.26	UBC9	0.047	UBC28	0.75	UBC9	1.57
	3	UBC9	0.388	UBC9	0.36	GAPDH	0.257	UBC9	0.75	UBC28	2.45
	4	UBC28	0.411	PPR	0.37	TIP41	0.354	PPR	0.8	GAPDH	4.16
	5	TIP41	0.457	GAPDH	0.95	UBC28	0.426	TIP41	0.8	PPR	4.47
	6	RH8	0.652	RH8	1.17	RH8	0.464	GAPDH	0.9	TIP41	6.24
	7	PPR	0.888	TIP41	1.2	PPR	0.493	RH8	1.23	RH8	6.74
*V. a*. f. *nigrum*	1	GAPDH	0.239	UBC9	0.07	GAPDH	0.014	UBC28	0.42	GAPDH	1.41
	2	UBC9	0.239	GAPDH	0.18	PPR	0.047	GAPDH	0.43	UBC9	2
	3	UBC28	0.251	UBC28	0.2	UBC28	0.133	UBC9	0.56	PPR	2.38
	4	PPR	0.284	PPR	0.34	CaCSa	0.136	PPR	0.57	UBC28	3
	5	TIP41	0.333	TIP41	0.42	TIP41	0.147	CaCSa	0.59	CaCSa	5.48
	6	CaCSa	0.378	CaCSa	0.55	UBC9	0.166	TIP41	0.61	TIP41	5.48
	7	RH8	0.432	RH8	0.64	RH8	0.178	RH8	0.67	RH8	7
Total	1	GAPDH	0.487	UBC28	0.19	UBC9	0.08	UBC28	0.59	UBC9	1.19
	2	UBC9	0.512	UBC9	0.25	CaCSa	0.104	CaCSa	0.62	UBC28	1.57
	3	CaCSa	0.571	PPR	0.47	GAPDH	0.125	GAPDH	0.66	CaCSa	3.13
	4	UBC28	0.599	CaCSa	0.59	TIP41	0.146	TIP41	0.72	PPR	3.46
	5	PPR	0.639	GAPDH	0.68	UBC28	0.167	UBC9	0.74	GAPDH	5
	6	TIP41	0.68	TIP41	0.78	PPR	0.173	PPR	0.75	TIP41	6
	7	RH8	0.701	RH8	0.98	RH8	0.195	RH8	0.99	RH8	7

*M* average of stability expression values, *SD* standard deviation of comparative ΔCq, *SV* stability value, *R* Pearson’s correlation, *GM* geometric mean.

#### NormFinder analysis

The expression stability of the seven candidate genes was analyzed using NormFinder, which is an excel based mathematical tool that measures gene expression stability by comparing the variation within and between user-defined sample groups. NormFinder ranks the control genes on the basis of their stability value (SV), lower value indicates higher gene expression stability and vice versa. The NormFinder algorithm results agreed with GeNorm analysis (Table 2). NormFinder selected *CaCSa* (SV = 0.043) and *UBC9* (SV = 0.047) as the most stable genes for *V. myrtilloides*, whereas, *GAPDH* (0.014) and *PPR* (0.047) for *V. a*. f. *nigrum*. For overall analysis, *UBC9* (0.080) and *CaCSa* (0.104) exhibited the most stable genes and *RH8* the least stable one (0.195).

#### BestKeeper analysis

BestKeeper ranks the stabilities of the candidate reference genes based on their standard deviation (SD) and the coefficient of variation (CV). Genes with SD > 1 were considered unacceptable reference genes. Based on the results from the BestKeeper analysis for *V. myrtilloides*, all genes except RH8 were calculated to have an SD value lower than 1 (Table 2). The rankings by BestKeeper analysis for *V. myrtilloides* showed that the most stable reference genes were *CaCSa* (0.62) followed by *UBC28* (0.75) and *UBC9* (0.75). For *V. a*. f. *nigrum UBC28* (0.42) and *GAPDH* (0.43) were observed as the most stable ones (Table 2). In the total dataset, *UBC28* and *CaCSa* were the most stable genes, with SD values of 0.59 and 0.62 respectively (p < 0.001). For all the three groups *RH8* exhibited the least stability.

#### ΔCq analysis

The ranking order of the seven candidate genes evaluated using ΔCq method were listed in Table 2. For *V. myrtilloides*, *UBC28* and *CaCSa* ranked as the most two stable RGs, whereas, for *V. a*. f. *nigrum UBC9* and *GAPDH* ranked as the top two. For the entire dataset, the results were similar to geNorm and NormFinder, with *UBC9* as the consistently stable reference gene, only differing in the ranking order. RH8 ranked as the least stable gene as demonstrated by other algorithms.

#### RefFinder analysis

**Figure 3:**
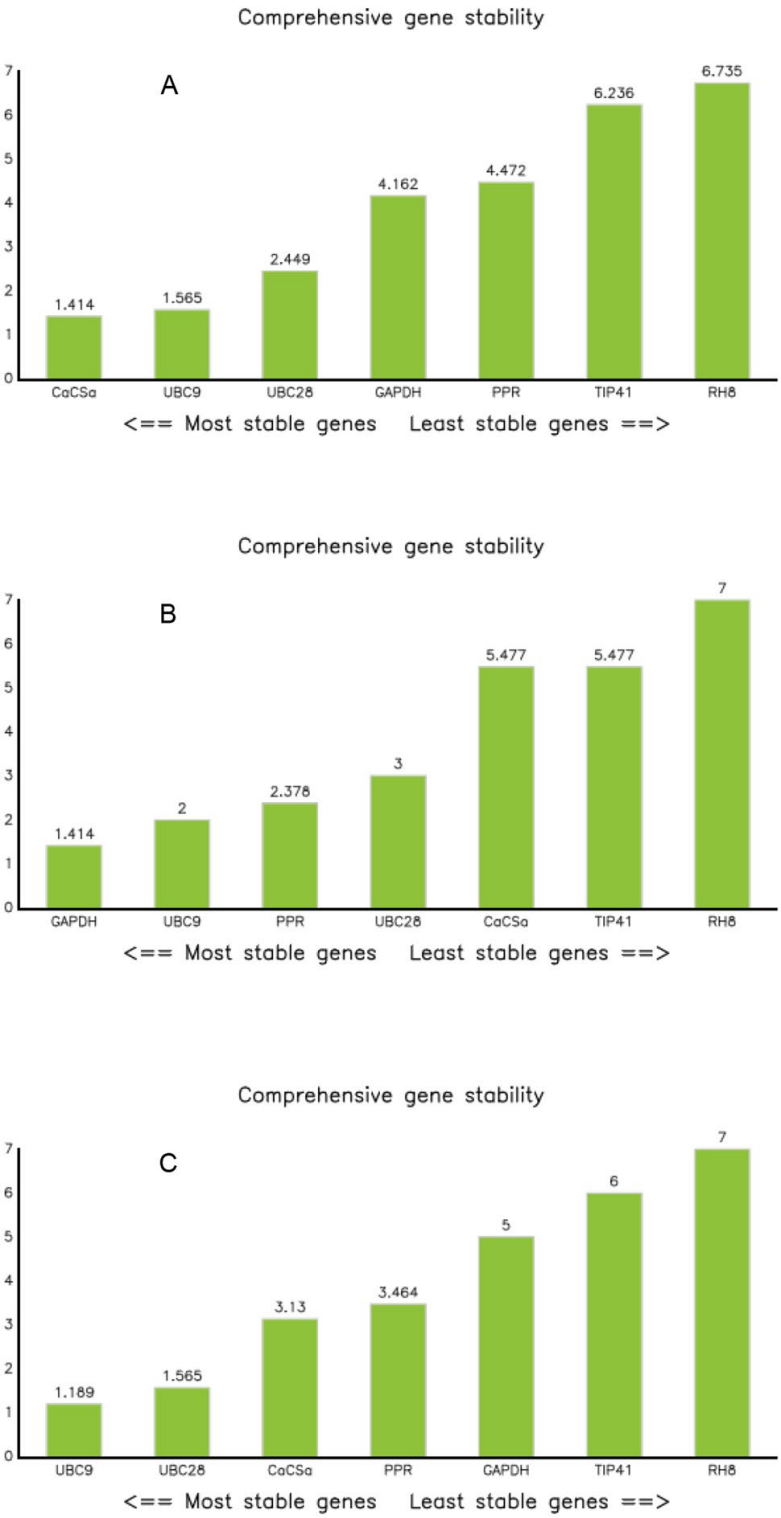
Comprehensive ranking of the candidate reference genes. **(A)** *V. myrtilloides*, **(B)** *V. a*. f. *nigrum* and **(C)** total samples. The expression stability was evaluated with the RefFinder tool to determine the overall comprehensive ranking order for each candidate reference gene.

A comprehensive ranking was performed to confirm the stability ranking of the seven candidate reference genes. In the comparative analysis of reference genes from *V. myrtilloides* and *V. a.* f*. nigrum*, *UBC9* and *UBC28* were ranked as the most stable RGs (Fig. 3). However, due to the differences in ploidy as well as defense response level, a comprehensive ranking order of reference genes was generated for each phenotype. The expression of *CaCSa*, *UBC9* and *UBC28* were found to be the most stable for gene expression normalization in *V. myrtilloides*. While, *GAPDH*, *UBC9* and *PPR* ranked as the best for *V. a*. f. nigrum. By contrast, *RH8* and *TIP41* ranked as the least stable genes for both the phenotypes.

### Validation of the best and least ranked reference genes

To validate the effectiveness of the selected reference genes, the expression pattern of *PR3* (MK292725) was analyzed in wild blueberry-Monilinia pathosystem. The relative transcript abundances of *PR3* gene was normalized to two most stable genes (*UBC9* and *GAPDH*) for *V. a*. f*. nigrum* and (*UBC9* and *UBC28*) for *V. myrtilloides* as resulted from the comprehensive analysis.

**Figure 4:**
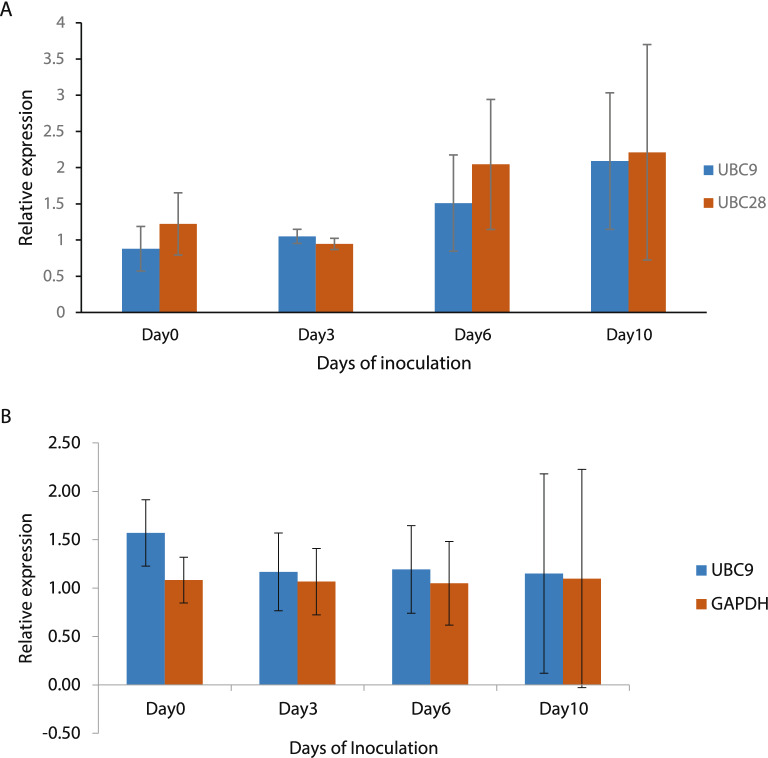
Relative expression of *PR3* gene at four time points (0, 3, 6 and 10 days) after inoculation using *M. vaccinii-corymbosi*. **(A)** *V. myrtilloides* normalized using the two most stable reference gene (*UBC9* and *UBC28*) and **(B)** *V. a*. f. *nigrum* normalized using the stable reference genes (*UBC9* and *GAPDH*). Values represent the means ± standard errors, where n = 3 biological replicates (with each replicate comprising tissue pooled from 15 stems).

In *V. myrtilloides*, the tolerant phenotype, data normalizations using the two most stable reference genes (*UBC9* and *UBC28*) resulted in consistent *PR3* expression pattern with gradual increase in expression over time points (Fig. 4A). However, in *V. a. f. nigrum*, the susceptible phenotype, the expression was high at day 0, then reduced the expression and not significantly elicited compared to control.

The expression data was also normalized using the least stable gene (*RH8*) reported for the two phenotypes (Fig. 4B). For both *V. a. f. nigrum* and *V. myrtilloides* no remarkable expression observed for all the analysed timepoints. These results indicated that the least stable gene, *RH8* failed to standardize the expression data effectively. Our results confirm that using different reference genes for normalisation causes great differences among the expression patterns.

## Discussion

RT-qPCR has emerged as a powerful tool to study transcript abundance of a specific gene in distinct biological samples owing to its precision, accuracy and sensitivity^11^. However, accurate normalization of gene expression remains a major criterion during qPCR analysis, as various steps during qPCR analysis can introduce variations arising from RNA extractions, cDNA synthesis, PCR procedure and sample loading^13^. Using a stably expressed reference gene is a prerequisite to obtain accurate interpretation of the transcript abundance results. Ideally, a reference gene should have constant expression in samples irrespective of the experimental conditions, developmental stages or species^22,23^. Several studies have reported the observation of variable expression of traditional reference genes in different plant species, as they have differential expression under different experimental conditions^24–26^. For example, Czechowski et al.^27^ reported that Arabidopsis have demonstrated variations in reference gene stability under different experimental conditions. Thus, the most appropriate reference genes should be properly evaluated and confirmed in all biological samples.

Many reliable reference genes have been determined in *Vaccinium* spp^16,17,28^ however, this study is the first to assess reference genes in wild blueberry-monilinia pathosystem. Also, the uniqueness of this study is that it was performed under field conditions. Field conditions differ from controlled environmental conditions, as plants are exposed to adverse environmental factors and multiple stresses. As stated by Samarth and Jameson^29^, the selection of appropriate reference genes from field conditions is more complex than controlled environmental studies. Tashiro et al.^30^ also pointed out the necessity of reference gene validation in studies involving non-model plant species from heterologous plant population. In this study, we assessed seven RGs for their use as internal controls in gene expression studies of the wild blueberry phenotypes upon monilinia blight infection under field conditions.

In the present study, four algorithms (geNorm^8^, NormFinder^18^, BestKeeper^19^ and delta CT^20^) was used to evaluate the best suited reference gene in wild blueberry phenotypes, *V. myrtilloides* and *V. a*. f*. nigrum*, for studying gene expression pattern during Monilinia blight infection. Several studies indicated the use of multiple statistical algorithms which not only minimize the errors associated with reference gene selection but ensures a more reliable evaluation^31–34^. This is probably due to the differences in algorithm programs exhibited by each method^23,33^. Moreover, variability in the stability ranking of reference genes based on algorithms has been reported in several studies^35,36^. Interestingly, the ranking order generated by using four programs roughly the same for both the phenotypes, with the first three most stable genes differing only on the ranking order. In addition, the pairwise variation determined using geNorm could be used as an indicator for estimating the optimal number of reference genes for normalization. In this study, the pairwise variation values for both *V. myrtilloides* and *V. a*. f. *nigrum* was V_2/3_ < 0.15, indicates that two reference genes were sufficient for gene expression normalization.

Even though the ranking order of the candidate reference genes may have differed, all the statistical programs showed *UBC9* as the most stable reference gene in the analysed phenotypes having different ploidy level. In this study, *GAPDH* and *UBC9* were observed as most stable reference genes for *V. angustifolium*. f. *nigrum* and *UBC9*, *UBC28* and *CaCSa* for *V. myrtilloides*. *GAPDH* was used for normalization in studies with *V. myrtillus*- Botrytis pathosystem^37^. Vashisth et al.^16^ and Die and Rowland^17^ ranked reference genes on vegetative and reproductive organs of rabbiteye and southern highbush blueberry. Vashisth et al.^16^ reported *CaCSa*, *RH8*, and *UBC28* as the most stably expressed gene in southern highbush blueberry across multiple organs analysed. Die and Rowland^17^ also reported *RH8*, *CaCSa*, *PPR*, *GAPDH* and *UBC9* (M = 0.483 and CV = 0.210) as the most stably expressed reference genes for floral bud tissues evaluated in highbush blueberry. However, in our study, *RH8* and *TIP41* were found to be the least stable reference gene for all the assessed phenotypes. Several studies have reported variable expression levels in reference genes among closely related species^38^. Moreover, several studies demonstrated variations in expression profiles of reference genes in different pathosystems^33,39^.

*PR3* was used as a target gene to validate the credibility of the selected reference genes. *PR3* which include chitinases of classes Ia, Ib, II, IV, VI, and VII are important weaponry of plants against pathogens^40^. According to Thomma et al.^41^, *PR3* which belong to the pathogenesis-related (*PR*) protein family play an important role in plant defense response to necrotrophic pathogens. Several studies reported the up-regulation of PR genes against many phytopathogenic fungi^42–44^. When comparing the most stable and least stable reference genes, the expression of target gene was consistent and upregulated in *V. myrtilloides* (*UBC9* and *UBC28*) and *V. a*. f. *nigrum* (*UBC9* and *GAPDH*) even though differences in expression observed between the phenotypes. However, no response observed in both phenotypes when analysed using the least stable gene (*RH8*). Our results agree with the findings of Cardot et al.^45^, where elicitation of chitinase genes observed in tolerant than in susceptible varieties.

In conclusion, this is the first study in which a set of candidate reference genes was analysed in terms of their expression stability in wild blueberry phenotypes infected with *Monilinia vaccinii-corymbosi*. Five different statistical algorithms showed slight differences in the final ranking of reference gene, however by combining and analysing the data together, we demonstrated that *UBC9* is the most stably expressed transcript in wild blueberry phenotypes regardless of ploidy level.

## Methods

### Plant material, *M.vc* inoculations and experimental design

The wild blueberry diploid (*V. myrtilloides*) and tetraploid (*V. angustifolium* f. *nigrum*) phenotypes were selected from a commercial wild blueberry field, NS, Canada. Three biological replicates were selected for each phenotype and each replicate was separated into two, 0.5 × 1 m sample areas. One day before inoculation, one sample area within each replicate was sprayed with the fungicide Proline (a.i. prothioconazole) at a rate of 315 ml product·ha^-1^ using a CO_2_ powered, Bell spray Inc. hand-held research sprayer with 2 m boom with 4 Tee Jet Visiflow 8003VS nozzles at a pressure of 220 kPa to serve as control plots. *Monilinia vaccinii-corymbosi* inoculum was prepared from four-week old *M.vc* cultures isolated from monilinia blighted shoots and mummy berries. Floral buds at F3 stage (floral bud scale separation and appearance of new growth) was tagged for each phenotype and inoculum (2 × 10^5^ ascospores·mL^−1^) was sprayed at all angles until runoff. Each phenotype sample area was immediately covered with 2 mm plastic film and row cover to provide incubating conditions (100% RH), which created conditions required for Monilinia infection^46^. After 72 h, the plastic film and row cover were removed and floral bud tissue from 15 random stems in each plot (control and inoculated) was harvested for RNA extraction and immediately flash frozen in liquid and stored at − 80 °C until further use. Floral bud tissues were collected as day 0 (before inoculation), 3, 6 and 10 days after inoculation.

### Total RNA isolation and cDNA synthesis

Total RNA was isolated from *V. angustifolium* f. *nigrum* and *V. myrtilloides* floral buds inoculated with *M.vc* as well as from control buds using RNeasy plant mini kit (Qiagen, US) following manual instructions. Residual genomic DNA was digested by RNase-free DNase (Qiagen, US) according to the manufacturer’s instructions. The concentration and purity of RNA samples was assessed using Nanodrop ND 1,000 spectrophotometer. RNA samples with an OD260/280 value (1.9–2.1) and OD260/230 (≥ 2.0) was used to determine the quality and purity of the extracted RNA^34^. RNA integrity was assessed by using 1.2% (w/v) agarose gel electrophoresis. cDNA was synthesized from 1 µg of total RNA in a final reaction volume of 20 µl using High Capacity cDNA Reverse Transcription Kit (Applied Biosystems) according to the manual instructions and stored at − 20 °C until use.

### Candidate reference genes: selection, primer design and amplification efficiency

Seven candidate genes were selected based on previous studies on *V. corymbosum* (highbush blueberry)^16^ and *V. myrtillus* (European blueberry)^37^ to identify the most suitable reference genes for gene expression analysis in wild blueberry. *V. corymbosum* ESTs (https://www.vaccinium.org) were mined to design primers for *CaCSa*, *UBC9*, *TIP41* and *PPR* using Primer Premier 5.0 (Premier Biosoft International, Palo Alto, California, USA) (Table 1). The primer sequences were blasted on NCBI database (https://blast.ncbi.nlm.nih.gov) to determine their homology with respective genes. A cDNA pool representing all the samples per phenotype was used to determine the amplification efficiency of each target/reference gene^31^. A tenfold cDNA dilution series (10, 10^2^, 10^3^, 10^4^, and 10^5^) was used to generate a standard curve for estimation of amplification efficiency (E = (10^[−1/slope]^ − 1) × 100%) and correlation coefficient (R^2^)^31^.

### Quantitative real-time PCR (qPCR)

qPCR assay was performed using a CFX Connect Real-time Detection System (Biorad, US) to analyze the specific expression of reference/target gene. Each PCR reaction mixture (10 µl) contained 2 µl of diluted cDNA (20-fold dilution), 5 µl SsoAdvanced SYBR Green Supermix (Biorad), and 1 µl (10 nM) of each forward and reverse primer. The amplification conditions were as follows: an initial denaturation at 95 °C for 180 s, followed by 40 cycles at 95 °C for 10 s, 60 °C for 20 s. Each run was completed with a melting curve analysis (65–95 °C with at increments of 0.5 °C) to verify the specificity of the amplification. The cycling conditions were based on the method described by Petriccione et al.^33^. A no-template control (NTC) was included for each gene assay to confirm the absence of non-specific products^47^.

### Determination of reference gene expression stability

The Cq value (quantification value) of each reference gene under the four different time points for both *V. myrtilloides* and *V.a.*f *nigrum* was recorded using the qPCR system. Four widely used software: geNorm^8^, NormFinder^18^, BestKeeper^19^, and ΔCq^20^ method was used to rank the expression stability of the reference genes. Finally, we used RefFinder^21^, a web-based user-friendly comprehensive tool, which integrates all four algorithms providing an overall ranking of the used genes.

The GeNorm algorithm, which is a module of qbase + software package (Biogazelle), was used to evaluate the candidate reference genes based on their expression stability values (M-values) and pairwise variations (Vn/Vn + 1)^48^. The default set value was 1.5; gene with the lowest M-value was the most stably expressed one. The computed pairwise variation (Vn/n + 1), was used to determine the optimal number of reference genes required for normalisation of the data. A (Vn/n + 1) value < 0.15 indicated the appropriate number of reference genes required for analysis^8^.

For Normfinder, the raw Cq values were converted into relative quantities (RQ) using the formula RQ = 2^(Cq min − Cq sample)^, where Cq min is the lowest Cq value across the sample pool. Normfinder evaluated the expression stability of candidate reference genes at inter-group and intra-group levels. Ideally, the two genes with the lowest stability values were the most appropriate genes to be used for normalisation^18^.

BestKeeper was performed using the original Microsoft Excel-based formulas^19^. It calculates the standard deviation of the Cq value between the whole data set, and the gene with the lowest standard deviation (SD) is proposed as most suitable. The comparative Δ*Cq* method manually compares relative expression of pairs of genes within each sample.

### Validation of reference genes

To confirm the reliability of selected reference genes, the relative expression profiles of *PR3* gene was determined and normalized with the two most stable and two least stable genes. The relative expression levels were calculated by 2^−△△Ct^ method^49^. For each qPCR experiment, three technical replicates were performed for each biological replicate. A one-way analysis of variance (ANOVA) was performed using the PROC GLIMMIX procedures of SAS (version 9.3, SAS institute, Inc., Cary, NC) for each time-point. Fisher’s LSD was used for multiple means comparison at the level of α = 0.05.

## Supplementary information

Supplementary Information.

## Data Availability

The data that support the findings of this study are available in the article and Supplementary Files.

## References

[1] Hildebrand PD, Braun PG (1991). Factors affecting infection of lowbush blueberry by ascospores of *Monilinia vaccinii-corymbosi*. Can. J. Plant Pathol..

[2] Percival, D., Jose, S., Guo, L., Schilder, A. & Olson, R. A. *Monilinia vaccinii-corymbosi* sensitivity to demethylation inhibitor fungicides and its effect on Monilinia blight control in wild blueberry fields. In *North American Blueberry Research and Extension Workers Conference*. Vol. 18 (2018). https://digitalcommons.library.umaine.edu/nabrew2018/proceedingpapers/proceedingpapers/18.

[3] Janes DE, Percival DC (2003). Trends in lowbush blueberry cultivar development. J. Am. Pomol. Soc..

[4] Galletta, G. J. Blueberries and cranberries. In *Advances in Fruit Breeding* (eds. J. Janick, J. N. Moore) 154–196 (Purdue University Press, West Lafayette, 1975).

[5] Ehlenfeldt, M. & Stretch, A. Resistance to blighting by *Monilinia vaccinii-corymbosi* in diploid and polyploid vaccinium species. *HortScience* **36**. 10.21273/HORTSCI.36.5.955 (2001).

[6] Morrison S, Smagula JM, Litten W (2000). Morphology, growth, and rhizome development of *Vaccinium angustifolium* Ait. seedlings, rooted softwood cuttings, and micropropagated plantlets. HortScience.

[7] Wise RP, Moscou MJ, Bogdanove AJ, Whitham SA (2007). Transcript profiling in host–pathogen interactions. Annu. Rev. Phytopathol..

[8] Vandesompele J (2002). Accurate normalization of real-time quantitative RT-PCR data by geometric averaging of multiple internal control genes. Genome Biol..

[9] Gachon C, Mingam A, Charrier B (2004). Real-time PCR: What relevance to plant studies?. J. Exp. Bot..

[10] Kozera B, Rapacz M (2013). Reference genes in real-time PCR. J. Appl. Genet..

[11] Bustin SA (2002). Quantification of mRNA using real-time reverse transcription PCR RT-PCR: Trends and problems. J. Mol. Endocrinol..

[12] Radonić A (2004). Guideline to reference gene selection for quantitative real-time PCR. Biochem. Biophys. Res. Commun..

[13] Huggett J, Dheda K, Bustin S, Zumla A (2005). Real-time RT-PCR normalisation. Strategies and considerations. Genes Immun..

[14] Dheda K (2004). Validation of housekeeping genes for normalizing RNA expression in real-time PCR. Biotechniques.

[15] Yang Q (2014). Reference gene selection for qRT-PCR in *Caragana korshinskii* Kom. under different stress conditions. Mol. Biol. Rep..

[16] Vashisth T, Johnson L, Malladi A (2011). An efficient RNA isolation procedure and identification of reference genes for normalization of gene expression in blueberry. Plant Cell Rep..

[17] Die JV, Rowland LJ (2013). Superior cross-species reference genes: A blueberry case study. PLoS ONE.

[18] Andersen CL, Jensen JL, Ørntoft TF (2004). Normalization of real-time quantitative reverse transcription-PCR data: A model-based variance estimation approach to identify genes suited for normalization, applied to bladder and colon cancer data sets. Cancer Res..

[19] Pfaffl MW, Tichopad A, Prgomet C, Neuvians TP (2004). Determination of stable housekeeping genes, differentially regulated target genes and sample integrity: BestKeeper—Excel-based tool using pair-wise correlations. Biotechnol. Lett..

[20] Silver, N., Best, S., Jiang, J. & Thein, S. L. Selection of housekeeping genes for gene expression studies in human reticulocytes using real-time PCR. *Bmc Mol Biol* **7** (2006). 10.1186/1471-2199-7-33 PMC1609175 17026756

[21] Xie F, Xiao P, Chen D, Xu L, Zhang B (2012). miRDeepFinder: A miRNA analysis tool for deep sequencing of plant small RNAs. Plant Mol. Biol..

[22] Wan H, Zhao Z, Qian C, Sui Y, Malik AA, Chen J (2010). Selection of appropriate reference genes for gene expression studies by quantitative real-time polymerase chain reaction in cucumber. Anal. Biochem..

[23] Kumar K, Muthamilarasan M, Prasad M (2013). Reference genes for quantitative real-time PCR analysis in the model plant foxtail millet (*Setaria italica* L.) subjected to abiotic stress conditions. Plant Cell Tissue Organ. Cult..

[24] Gutierrez L, Mauriat M, Pelloux J, Bellini C, Van Wuytswinkel O (2008). Towards a systematic validation of references in real-time RT-PCR. Plant Cell.

[25] Ma S, Niu H, Liu C, Zhang J, Hou C, Wang D (2013). Expression stabilities of candidate reference genes for RT-qPCR under different stress conditions in soybean. PLoS ONE.

[26] Zhu J, Zhang L, Li W, Han S, Yang W, Qi L (2013). Reference gene selection for quantitative real-time PCR normalization in *Caragana intermedia* under different abiotic stress conditions. PLoS ONE.

[27] Czechowski T, Stitt M, Altmann T, Udvardi MK, Scheible WR (2005). Genome-wide identification and testing of superior reference genes for transcript normalization in Arabidopsis. Plant Physiol..

[28] Zifkin M (2012). Gene expression and metabolite profiling of developing highbush blueberry fruit indicates transcriptional regulation of flavonoid metabolism and activation of abscisic acid metabolism. Plant Physiol..

[29] Samarth A, Jameson PE (2019). Selection of reference genes for flowering pathway analysis in the masting plants, *Celmisia lyallii* and *Chionochloa pallens*, under variable environmental conditions. Sci. Rep..

[30] Tashiro RM, Philips JG, Winefield CS (2016). Identification of suitable grapevine reference genes for qRT-PCR derived from heterologous species. Mol. Genet. Genomics.

[31] Monteiro F, Sebastiana M, Pais MS, Figueiredo A (2013). Reference gene selection and validation for the early responses to downy mildew infection in susceptible and resistant *Vitis vinifera* cultivars. PLoS ONE.

[32] Yang H (2014). Selection and evaluation of novel reference genes for quantitative reverse transcription PCR (qRT-PCR) based on genome and transcriptome data in *Brassica napus* L.. Gene.

[33] Petriccione M, Mastrobuoni F, Zampella L, Scortichini M (2015). Reference gene selection for normalization of RT-qPCR gene expression data from *Actinidia deliciosa* leaves infected with *Pseudomonas syringae* pv. *actinidiae*. Sci. Rep..

[34] Shivhare R, Lata C (2016). Selection of suitable reference genes for assessing gene expression in pearl millet under different abiotic stresses and their combinations. Sci. Rep..

[35] Andrade LM, Brito M, Peixoto Junior R (2017). Reference genes for normalization of qPCR assays in sugarcane plants under water deficit. Plant Methods.

[36] Ye J, Jin C, Li N (2018). Selection of suitable reference genes for qRT-PCR normalisation under different experimental conditions in *Eucommia ulmoides* Oliv. Sci Rep..

[37] Koskimäki JJ (2009). Flavonoid biosynthesis and degradation play a role in early defence responses of bilberry (*Vaccinium myrtillus*) against biotic stress. Eur. J. Plant Pathol..

[38] Jarosova J, Kundu J (2010). Validation of reference genes as internal control for studying viral infections in cereals by quantitative real-time RT-PCR. BMC Plant Biol..

[39] Borges A, Tsai S, Caldas D (2012). Validation of reference genes for RT-qPCR normalization in common bean during biotic and abiotic stresses. Plant Cell Rep..

[40] Rawat S, Ali S, Mittra B, Grover A (2017). Expression analysis of chitinase upon challenge inoculation to Alternaria wounding and defense inducers in *Brassica juncea*. Biotechnol. Rep..

[41] Thomma BPHJ (1998). Separate jasmonate-dependent and salicylate-dependent defense-response pathways in Arabidopsis are essential for resistance to distinct microbial pathogens. Proc. Natl. Acad. Sci. USA.

[42] Mishina TE, Zeier J (2007). Pathogen-associated molecular pattern recognition rather than development of tissue necrosis contributes to bacterial induction of systemic acquired resistance in Arabidopsis. Plant J..

[43] Kusajima M (2010). Suppressive effect of abscissic acid on systemic acquired resistance in tobacco plants. J. Gen. Plant Pathol..

[44] González-Grandío E, Poza-Carrión C, Sorzano CO, Cubas P (2013). BRANCHED1 promotes axillary bud dormancy in response to shade in Arabidopsis. Plant Cell.

[45] Cardot C (2019). Comparison of the molecular responses of tolerant, susceptible and highly susceptible grapevine cultivars during interaction with the pathogenic fungus *Eutypa lata*. Front. Plant Sci..

[46] Delbridge, R. & Hildebrand, P. *Monilinia Blight of Lowbush Blueberry*. https://cdn.dal.ca/content/dam/dalhousie/images/sites/wildblueberry/pdfs/Monilinia_Blight_Lowbush_Blueberry.pdf (1997).

[47] Li MY, Song X, Wang F, Xiong AS (2016). Suitable reference genes for accurate gene expression analysis in parsley (*Petroselinum crispum*) for abiotic stresses and hormone stimuli. Front. Plant Sci..

[48] Hellemans J, Mortier G, De Paepe A, Speleman F, Vandesompele J (2007). qBase relative quantification framework and software for management and automated analysis of real-time quantitative PCR data. Genome Biol..

[49] Livak KJ, Schmittgen TD (2001). Analysis of relative gene expression data using real-time quantitative PCR and the 2^−ΔΔCt^ method. Methods.

