# Supplementary material for: Selection and validation of reliable reference genes for gene expression studies from Monilinia vaccinii-corymbosi infected wild blueberry phenotypes
Source: Sci Rep. 2020 Jul 16;10:11688. doi: 10.1038/s41598-020-68597-9 (PMC7366731; doi:10.1038/s41598-020-68597-9)
Supplement: Supplementary file 1 — Supplementary Information. [file 41598_2020_68597_MOESM1_ESM.pdf]

Selection and validation of reliable reference genes for gene expression studies from *Monilinia vaccinii-corymbosi* infected wild blueberry phenotypes

Sherin Jose[a]*, Joel Abbey[a], Laura Jaakola[b,c] and David Percival[a]

[a] Wild Blueberry Research Program, Faculty of Agriculture, Dalhousie University, NS, Canada B2N 5E3

[b] Climate laboratory Holt, Department of Arctic and Marine Biology, The Arctic University of Norway, NO-9037 Tromsø, Norway

[c] NIBIO, Norwegian Institute of Bioeconomy Research, P.O. Box 115, NO-1431 Ås, Norway

*Corresponding author

Sherin Jose

[a] Wild Blueberry Research Program, Faculty of Agriculture, Dalhousie University, NS, Canada B2N 5E3

E-mail: s.jose@dal.ca

Supplementary Table S1. Raw Cq values obtained for each wild blueberry phenotype

		CaCSa	GAPDH	TIP41	UBC28	UBC9	PPR	RH8
V. myrtilloides	Day0-1	25.14	22.30	28.23	22.25	23.63	28.58	27.65
	Day0-2	23.89	20.61	26.99	21.35	22.74	27.18	26.17
	Day0-3	24.61	20.65	27.08	21.34	22.46	31.33	26.88
	Day3-1	26.61	23.53	30.17	24.72	25.08	27.83	28.58
	Day3-2	24.20	20.21	26.51	21.30	22.23	27.78	25.39
	Day3-3	23.73	19.93	26.26	20.87	21.73	27.36	25.23
	Day6-1	23.41	19.31	25.49	20.87	21.93	27.43	24.86
	Day6-2	24.22	19.98	26.41	22.15	22.48	28.52	25.66
	Day6-3	23.68	19.66	25.68	20.92	21.94	28.23	24.92
	Day10-1	24.22	20.88	26.13	21.64	23.36	29.09	26.35
	Day10-2	23.25	19.64	25.11	20.40	21.66	27.31	28.04
	Day10-3	24.00	19.52	25.71	21.23	21.84	27.76	25.22
V.a.f. nigrum								
	Day0-1	24.04	20.71	26.41	21.23	22.66	27.87	25.87
	Day0-2	25.21	21.67	27.27	22.36	23.57	29.11	27.25
	Day0-3	24.78	20.40	26.69	21.58	22.90	28.54	26.62
	Day3-1	23.94	20.65	25.59	21.07	23.09	27.35	25.68
	Day3-2	24.28	20.40	26.45	21.84	23.16	27.47	25.31
	Day3-3	23.15	18.93	25.08	20.54	25.07	26.63	24.54
	Day6-1	23.41	19.59	25.90	21.15	23.05	27.27	25.38
	Day6-2	22.88	19.10	25.21	21.03	22.08	26.87	24.56
	Day6-3	23.08	19.18	25.69	21.05	22.11	26.37	24.19
	Day10-1	23.57	19.36	25.67	20.10	22.50	27.36	25.24
	Day10-2	23.88	20.00	25.81	21.49	22.80	27.34	25.76
	Day10-3	23.00	19.25	24.73	20.88	21.96	26.56	24.81

Supplementary Figure S1. Melting curve analysis of seven candidate reference gene with individual single peak indicating the specificity of the amplifications.

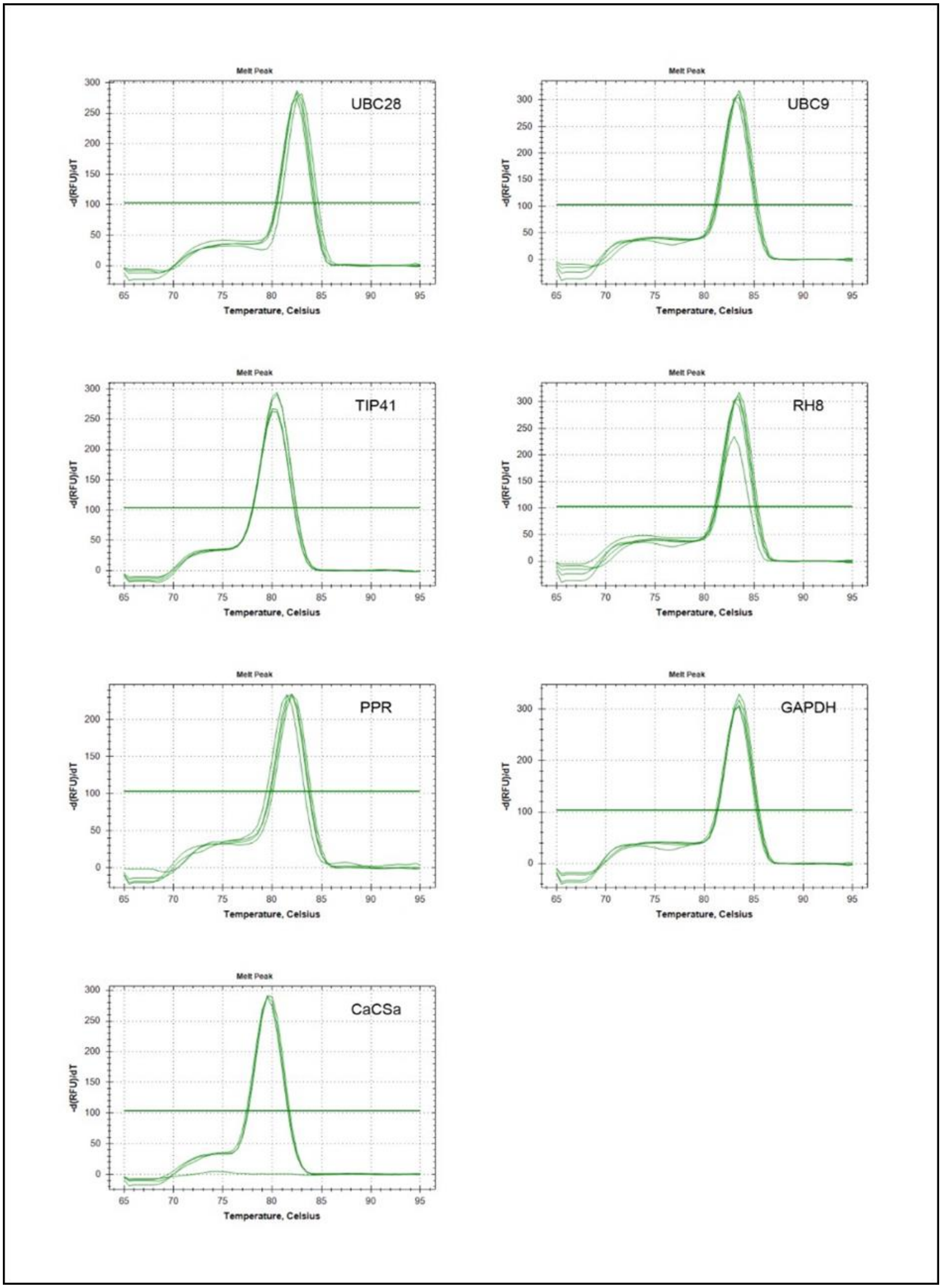

Supplementary Figure S2. Agarose gel electrophoresis (1.5%) showing amplified PCR products. 1- UBC9, 2- PPR, 3- GAPDH, 4- TIP41, 5- CaCSa, 6- UBC28, 7- RH8, along with 100bp ladder (M).

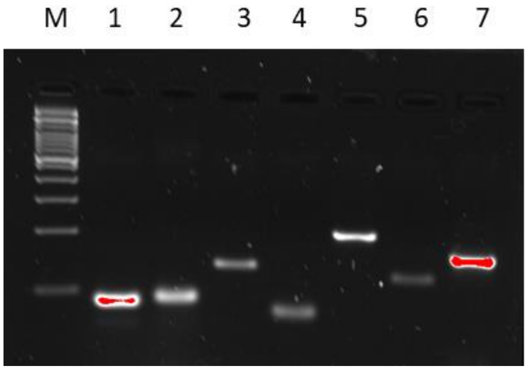

Supplementary Figure S3. Standard curves for the estimation of PCR efficiency obtained from seven candidate housekeeping genes. The linear correlation (R^2) and PCR efficiencies (E = (10[−1/slope] −1) × 100%)) were calculated from the standard curve.

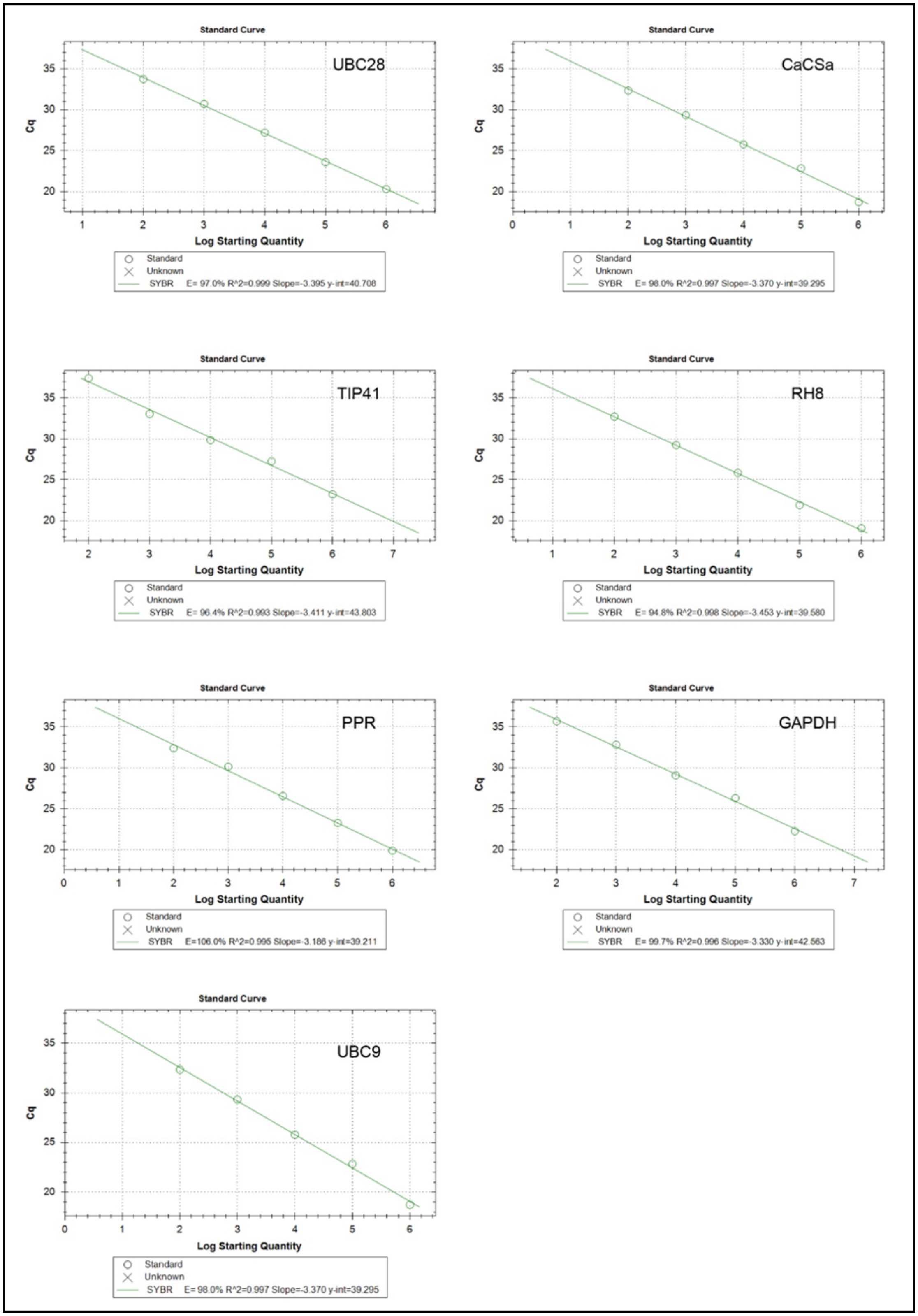
Standard Curve
UBC28
Cq
Log Starting Quantity
Standard
Unknown
SYBR E= 97.0% R^2=0.999 Slope=-3.395 y-int=40.708
Standard Curve
CaCSa
Cq
Log Starting Quantity
Standard
Unknown
SYBR E= 98.0% R^2=0.997 Slope=-3.370 y-int=39.295
Standard Curve
TIP41
Cq
Log Starting Quantity
Standard
Unknown
SYBR E= 96.4% R^2=0.993 Slope=-3.411 y-int=43.803
Standard Curve
RH8
Cq
Log Starting Quantity
Standard
Unknown
SYBR E= 94.8% R^2=0.998 Slope=-3.453 y-int=39.580
Standard Curve
PPR
Cq
Log Starting Quantity
Standard
Unknown
SYBR E=106.0% R^2=0.995 Slope=-3.186 y-int=39.211
Standard Curve
GAPDH
Cq
Log Starting Quantity
Standard
Unknown
SYBR E= 99.7% R^2=0.996 Slope=-3.330 y-int=42.563
Standard Curve
UBC9
Cq
Log Starting Quantity
Standard
Unknown
SYBR E= 98.0% R^2=0.997 Slope=-3.370 y-int=39.295
